# The Positive and Negative Affect Schedule — Food Allergy (PANAS-FA): Adaptation and psychometric properties^⋆^

**DOI:** 10.1016/j.waojou.2021.100615

**Published:** 2021-12-05

**Authors:** Gabriel Lins de Holanda Coelho, Aideen Byrne, Jonathan Hourihane, Audrey DunnGalvin

**Affiliations:** a University College Cork, Ireland; b Crumlin Children's Hospital, Ireland; c Royal College of Surgeons, Ireland; d School of Applied Psychology, University College Cork, Ireland

**Keywords:** Food allergy, Emotions, Affect, Allergy, Psychometrics

## Abstract

**Background:**

Food allergy (FA) is a worldwide concern, increasing up to 50% in the past decade, with a 700% rise in hospitalizations because of anaphylaxis. Individuals diagnosed with FA must have the emotional resources to cope with the many challenges that arise from self-management tasks and the social limitations that FA presents. Therefore, it is clear that close consideration of heightened emotions due to FA is needed.

**Method:**

The present research aimed to adapt the Positive and Negative Affect Schedule (PANAS), one of the most used questionnaires available to measure mood or emotion worldwide, for a population of individuals with FA. We performed one study (*N* = 205; *M*_age_ = 37.37; Age range = 18–72). To adapt the measure, we asked participants to what extent they “generally” felt about having a FA, through 20 items (eg, strong, alert – positive affect; upset, scared – negative affect). We used Item Response Theory, Confirmatory Factor Analysis (CFA), and reliability estimates to assess the data. We also propose a shorter version of the PANAS-FA, using its “best items”. Finally, we also used the General Anxiety Disorder-7 measure and Need for Affect Questionnaire to assess convergent validity.

**Results:**

The PANAS-FA presented a good model fit and strong item parameters. We removed 4 items from each factor for the shorter version, which presented difficulty levels slightly higher than recommended. The short PANAS-FA presented comparable results to the longer version. The measure also showed significant associations with general anxiety and need for affect, which assesses to what extent an individual likes to engage in emotion-inducing situations. Through a mediational model, negative affect significantly influenced general anxiety, partially influenced by the extent individuals avoid emotional situations.

**Conclusion:**

We are confident that the adaptation of the Positive and Negative Affect Schedule focused on food allergy (PANAS-FA) provides a novel opportunity to understand the intrinsic associations between emotions and living with FA. Identifying which FA emotions are related to these factors may be vital for future interventions, providing an environment that focuses or promotes these emotions to enhance individual well-being.

## Introduction

Food allergy (FA) is a worldwide concern with up to 50% increased prevalence in the past decade, with a 700% rise in hospitalizations because of anaphylaxis.^1^ According to the US Food & Drug Administration (FDA), FA alone is responsible for around 90% of all allergic reactions.^2^ In the United States, a survey with 40 443 US Adults found a self-reported prevalence rate of 10.8% for FA, suggesting that-approximately 1 in 10 US adults have some type of food allergy.^3^ In addition to the reported adverse impact on Food Allergy Quality of Life (FAQL) for all age groups and families, the condition also has economic costs for health systems. This cost ranged between 55 and 151 billion Euro per year in Europe alone.^4^

Allergen avoidance and emergency management require patients and caregivers to have the proper skills to manage allergic reactions. They must also have the emotional resources to cope with the many challenges that arise from daily self-management tasks, such as avoiding allergens, reading labels, and carrying the adrenaline auto-injector, together with the related social restrictions that FA presents. The impact of FA on the FAQL of patients and caregivers is highlighted in research, with results showing a decrease in FAQL related to daily tasks, and stress comparable to or greater than other paediatric chronic illness populations.^5^ One common outcome among FA patients and caregivers is the development of anxiety due to the constant fear about allergen exposure, allergic reactions, and fatal anaphylaxis, which drives poor FAQL.^6^^,^^7^^,^^8^^,^^9^ Therefore, it is clear that a closer consideration of heightened emotions because of food allergy is needed. A better assessment of how emotions influence the day-by-day lives of those affected by FA can help provide a more in-depth understanding of the impact of the disease on FAQL.

### Positive and Negative affect

Watson et al^10^ describe positive affect as representing to what extent people experience positive mood states, whereas negative affect represents the extent to which they experience harmful mood states. Their role has been extensively studied over the past decades. For instance, positive affect influences emotional well-being,^11^ besides attributing a feeling that life is essential.^12^ On the other hand, negative affect is negatively associated with emotional intelligence,^13^ and positively with depression.^14^ In chronic disease, emotions and coping mediate participation and shared medical decision-making in health care processes.^15^^,^^16^ Emotional demands deplete the resources needed for everyday self-care management of a condition, contributing to poor health outcomes. For example, emotional distress, coping strategies, and coping efficacy influence self-care behaviour and health outcomes in patients with diabetes mellitus.^15^ Moreover, maladaptive and negative emotions are strongly positively related to the psychological distress experienced by individuals with a chronic skin disease (ie, psoriasis, atopic eczema).^16^

The ability to capture the specific influence of positive and negative emotions can help us to better understand their role in FA, their associations with other variables, as well as allow us to develop initiatives to enhance the presence of positive affect. To date, no measure assesses emotions specific to FA. Typically, general emotion measures (eg, *Positive and Negative Affect Schedule* – PANAS) have been used in prior research. To provide a psychometrically adequate, reliable, and FA-specific tool to measure emotions, we describe here the development and validation of PANAS-FA (*Positive and Negative Affect Schedule – Food Allergy*), an adapted version of the original measure. We used several robust techniques (eg, item response theory, confirmatory factor analysis) to assure structure adequacy and reliability. Moreover, the associations to other measures (General Anxiety, Need for Affect) provide evidence of convergent validity to PANAS-FA. Finally, we assessed group differences regarding gender, auto-injector use (or not), and single vs multiple FAs. Finally, this research is part of the Food Allergy Coping and Emotions (FACES) project, aiming to understand better the underlying mechanisms of food allergy (ie, emotions, psychological responses).

## Method

### Participants and procedure

Participants were from the United Kingdom and were recruited through Prolific Academic (https://www.prolific.co/). On the platform, we applied several custom pre-screenings which accorded with study exclusion criteria, namely Diet Restriction (presenting a type of food allergy), together with the number of previous participations on Prolific (minimum of 25), and approval rate (minimum of 98%). To ensure that participants were properly reading and considering the questions, we included multiple “test items” within the questionnaires (eg, “*Please, select strongly disagree*). Ten participants failed to complete the multiple test items and were removed from any subsequent analyses. Two-hundred five individuals (*M*_age_ = 37.37; *SD*_age_ = 12.67; Age range = 18–72; Men = 63; Women = 142) completed the survey. Participants reported at least 1 type of food allergy (eg, peanuts = 58; shellfish = 45), had been diagnosed by a general practitioner∖family doctor (n = 93; 45.4%) or allergist (n = 42; 20.5%), with the majority diagnosed in 2005 or before (n = 114; 55.6%). For full information, please check Supplemental Table 1.

## Material

The *Positive and Negative Affect Schedule* (PANAS);^10^ is one of the most used questionnaires to measure mood or emotion. The scale is composed of 20 items, equally distributed among positive affect (eg, alert, determined, active) and negative affect (eg, distressed, scared, afraid). When answering, participants indicate to what extent they experienced each emotion in relation to a specific period of time (eg, present moment, past week, past year). The measure has shown good reliability levels (Cronbach's alpha ≥.84) over different time frames, besides being extensively assessed in diverse populations and using different temporal instructions.^17^ The measure was further validated in many countries, demonstrating cross-cultural applicability, including Brazil,^18^ Italy,^19^ Australia,^20^ Chile,^21^ and Canada.^22^ The PANAS measure is available in long-form (PANAS-X, 60 items);^23^ short form (I-PANAS-SF, 10 items);^24^, and in a version for children.^25^

In order to adapt the PANAS,^10^ we used gold standard methods to ensure the validity of the new FA-specific measure. Firstly, we made changes in its instructions. That is, instead of asking to what extent the respondent experienced emotions during a specific period, we asked them to indicate to what extent they “generally” felt about having a food allergy. This change was aimed to help respondents to rate emotions solely on their FA experience, instead of considering the influence of other daily situations. The 20 emotions remained the same, equally divided into positive affect (eg, *interested*, *alert*) and negative affect (eg, *distressed*, *ashamed*). Participants indicated their general feelings about FA using a five-point scale (1 = *Very slightly or not at all*; 5 = *Extremely*).

Secondly, we asked respondents to complete 2 additional well-validated measures to demonstrate convergent validity for the PANAS-FA. First, we used the *General Anxiety Disorder - 7* (GAD-7); ^26^ to assess anxiety. Participants have to indicate how often they are bothered by specific problems (eg, *Feeling nervous, anxious, or on the edge*) over the past 2 weeks, using a four-point response scale (1 = *Not at all sure*; 4 = *Nearly every day*). Secondly, to assess the need for affect, which represents how much individuals like to engage or avoid emotional situations, we used the *Need for Affect Questionnaire* – *Short Version*.^27^ Using a seven-point scale (−3 = *Strongly Disagree*; 3 = *Strongly Agree*), participants indicate their level of agreement to 10 items equally distributed into emotion approach (eg, *I feel that I need to experience strong emotions regularly*) and emotion avoidance (eg, *I find strong emotions overwhelming and therefore try to avoid them*). A higher score in the emotion approach dimension indicates a higher tendency to engage in emotional situations. In contrast, a higher score in emotion avoidance suggests a tendency to evade such events. In a FA context, individuals who are more likely to engage in situations that elicit strong emotions might be more likely to put themselves in risky situations.

### Data analysis

We used the “R”^28^ and JASP (https://jasp-stats.org/) to perform the analysis. In R, we used the Multidimensional Item Response Theory (*MIRT*) package,^29^ to assess item parameters (eg, discrimination, difficulty, and level of information) of the PANAS-FA. As the answer scale has more than 2 categories, we used the graded response model.^30^ Using the *lavaan* package,^31^ we performed multiple Confirmatory Factor Analysis (CFA), using the Diagonally Weighted Least Squares (DWLS) estimator. The following model fit indices were considered:^32^^,^^33^ For the (1) Comparative Fit Index (CFI) and (2) Tucker-Lewis Index (TLI), values over 0.90 are recommended; whereas for the (3) Root mean square error approximation (RMSEA), results should be lower than 0.80. To assess internal consistency (reliability), we used the *userfriendlyscience* package.^34^ We considered McDonald's omega (ω) and Cronbach's alpha (α), with recommended values over 0.70.^35^ In JASP, we performed multiple Pearson's r correlations, to assess convergent validity. Further, we also performed a mediation, using the maximum likelihood estimator and 5000 bootstrap simulations. Finally, we also performed t-tests to assess group differences.

## Results

### Item response theory (IRT)

First, we assessed items' discrimination, difficulty, and information levels, using IRT. Health outcomes researchers are increasingly applying IRT methods to questionnaire development and refinement, since it is a powerful tool that can result in precise, valid, and relatively brief instruments that minimize response burden.^36^ The results for discrimination and difficulty can be seen in Table 1. A highly discriminative item helps differentiate individuals with different latent trait levels — in this case, positive and negative emotions. To assess the discrimination levels (represented by an *a* on Table 1) of the PANAS-FA items, we used Baker's^37^ thresholds. For the positive affect factor, 8 items presented very high discrimination levels (a >1.7), and 2 presented high level (a between 1.35 and 1.69). For the negative affect factor, all items presented very high discrimination levels.

**Table 1:** Discrimination and difficulty parameters of the PANAS-FA.

	a	b_1_	b_2_	b_3_	b_4_	*b*(*m*)
*Positive*						
Interested	2.286	0.013	0.645	1.415	2.648	1.18
Excited	2.138	1.441	1.99	2.685	3.128	2.31
Strong	2.565	0.326	0.937	1.725	2.536	1.38
Enthusiastic	2.528	0.859	1.473	2.234	2.892	1.86
Proud	1.893	1.215	1.831	2.31	3.613	2.24
Alert	1.658	−1.023	−0.204	0.532	1.79	0.27
Inspired	2.715	0.866	1.504	2.08	2.831	1.82
Determined	3.139	−0.067	0.512	1.19	2.218	0.96
Attentive	1.609	−0.76	0.01	0.852	1.883	0.5
Active	2.755	0.131	0.645	1.517	2.375	1.17
*Negative*						
Distressed	2.62	−0.006	0.918	1.543	2.774	1.31
Upset	2.419	−0.085	0.916	1.466	2.92	1.3
Guilty	1.915	0.73	1.689	2	3.254	1.92
Scared	2.738	−0.134	0.757	1.48	2.088	1.05
Hostile	2.397	0.495	1.21	1.847	2.677	1.56
Irritable	2.245	−0.129	0.644	1.439	2.141	1.02
Ashamed	2.339	0.745	1.496	1.927	2.78	1.74
Nervous	2.901	−0.382	0.591	1.245	2.18	0.91
Jittery	1.996	0.281	1.271	1.835	2.979	1.59
Afraid	2.781	−0.122	0.74	1.357	2.124	1.02

*Note*: *a =* discrimination, *b =* difficulty

The difficulty level, within a survey, represents how much of the latent trait a person needs to hold to select the following superior option in the categories available in the answer scale. In this case, if an item of the PANAS-FA is seen as “too easy”, it means that most individuals (independently of their level in the latent trait) are likely to agree with it strongly. On the other hand, if the item is “too difficult”, only individuals with a higher level in the latent trait would strongly agree with it. Thus, Rauthmann^38^ recommends the items to be within a threshold level (eg, means across b1-b4 between −1.5 and 1.5) to present an adequate difficulty level. As shown by the means column *b*(*m*) on Table 1, 4 items of each factor (eg, excited, proud, guilty, hostile) were not within this recommended threshold.

**Fig. 1:**
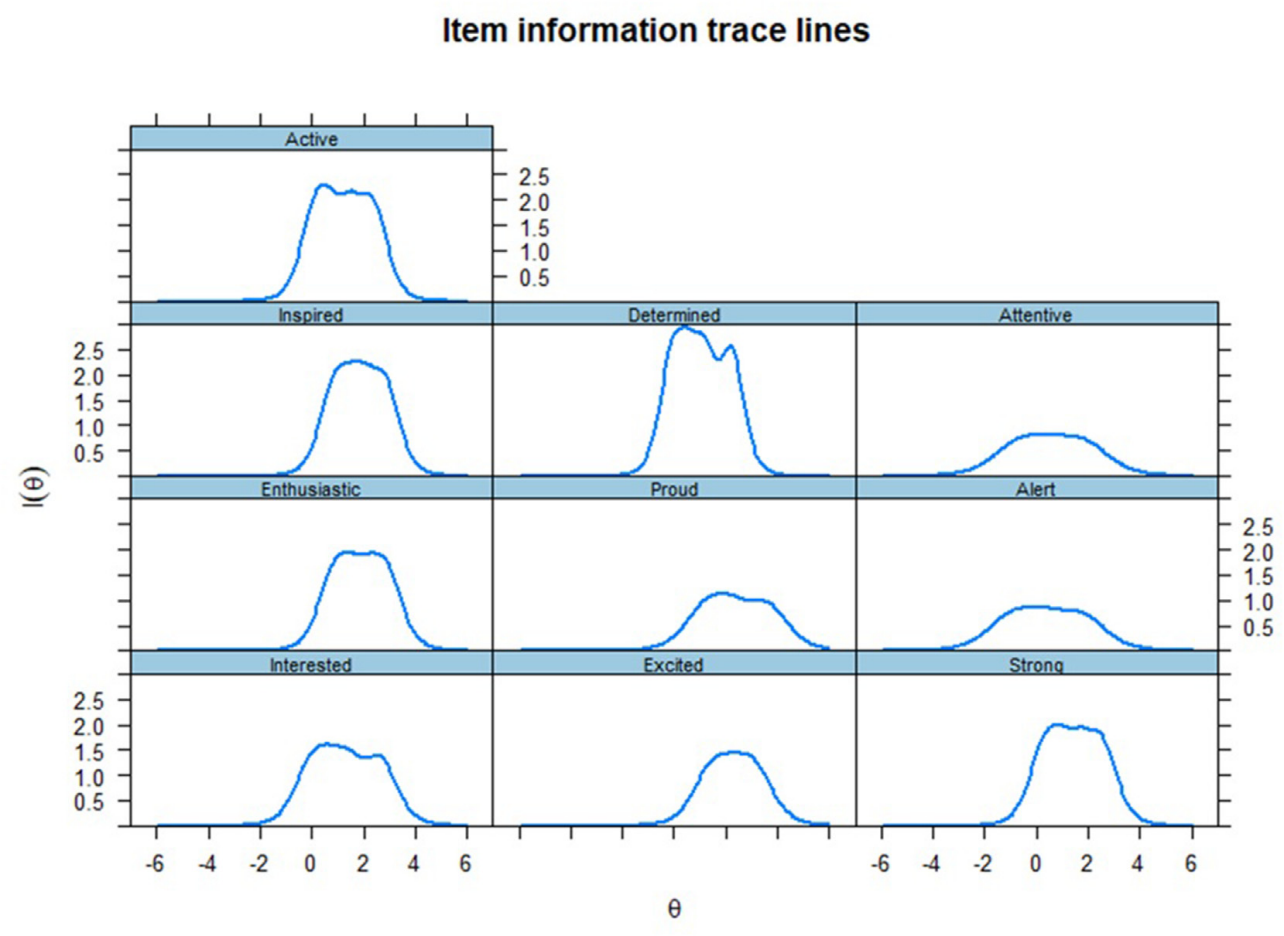
Item information curves for the positive emotions factor.

**Fig. 2:**
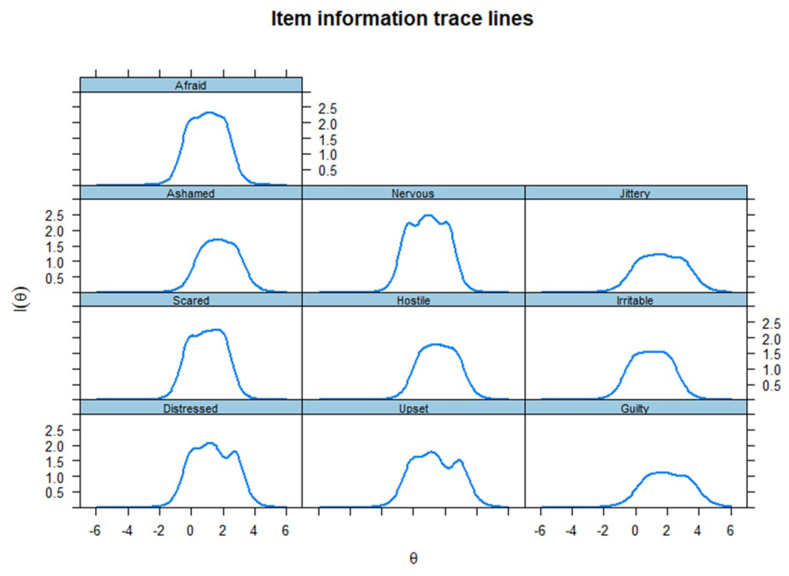
Item information curves for the negative emotions factor.

**Fig. 3:**
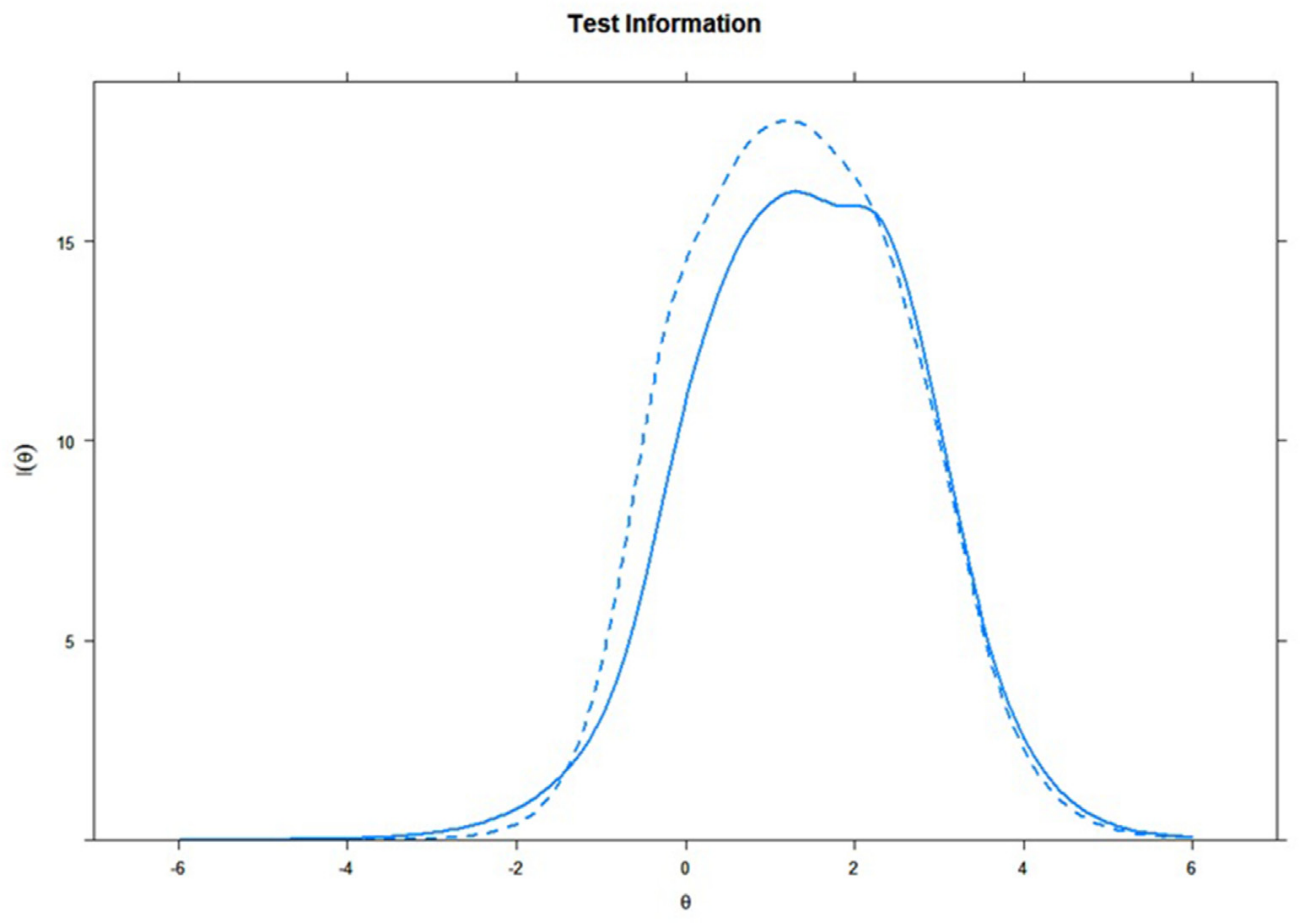
Test information curve of the PANAS-FA.

Finally, we assessed how much information an item can provide individually to its factor (Item Information Curve, ICC; Fig. 1, Fig. 2;^39^), and their summed information (Test Information Curve, TIC; Fig. 3). A bell-shaped curve indicates a good amount of information, whereas flat lines indicate no considerable information shared.^40^ As shown in Fig. 1, Fig. 2, the items (individually) contributed considerable information to their related factor. The summed of information of both positive and negative affects resulted in informative factors (Fig. 3). More informative factors are also more reliable, with total information of 10 presenting similar results to an internal consistency (ie, Cronbach's alpha) of 0.90.^41^

### Confirmatory factor analysis

**Fig. 4:**
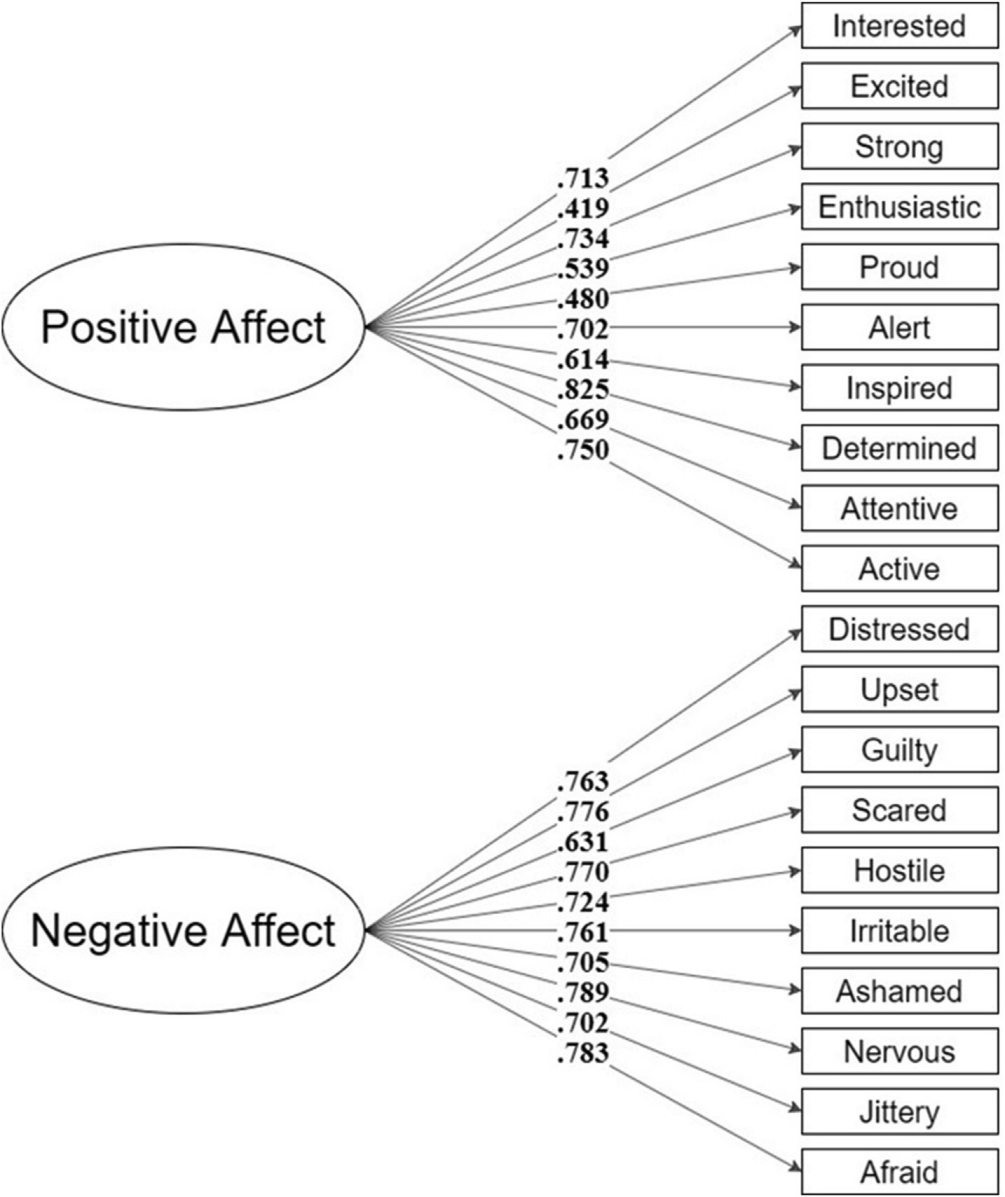
Long structure – PANAS-FA.

**Fig. 5:**
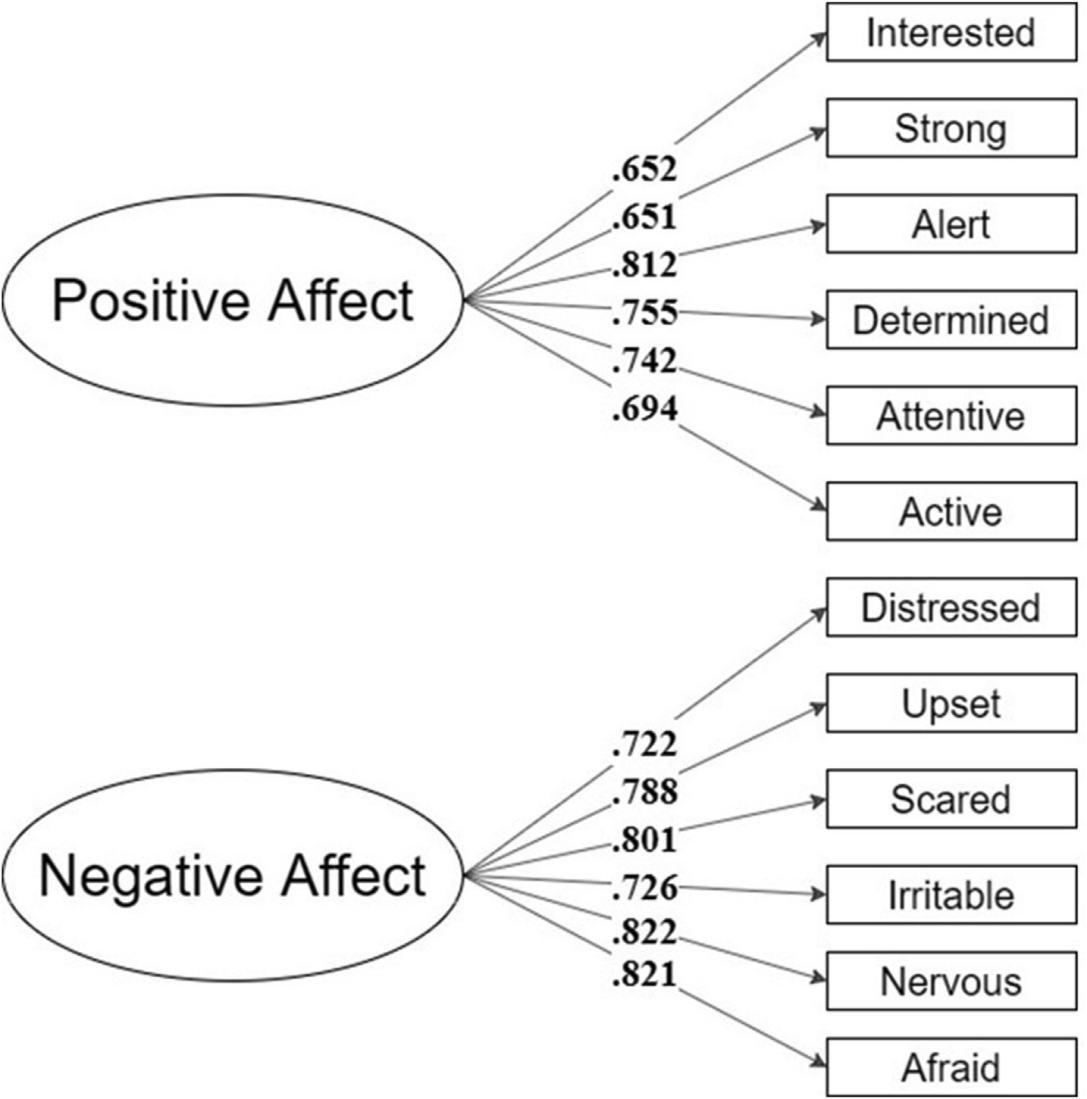
Short structure – PANAS-FA.

The item parameters provided by the Item Response Theory output showed 4 items from each factor that presented a difficulty level over the recommended threshold.^38^ These items were “too difficult”, indicating that most participants were “strongly disagreeing” with them. When assessing the content of these items, such findings seem reasonable and meaningful. For instance, it is unlikely that an individual with FA will feel enthusiastic (positive affect) about living with FA. Therefore, we decided to assess the fit for 2 different models, one considering the long measure, with 10 items per factor, and 1 considering only the items with a recommended difficulty level (6 items per factor). For that, we performed two CFAs, using the DWLS estimator. Results indicated a good model fit for both versions: Long, CFI = .98; TLI = .98, RMSEA = .038 [IC90% .021-.052]; Short, CFI = .99; TLI = .98, RMSEA = .043 [IC90% .011-.066]. All the factorial weights (lambdas) were statistically different from zero (λ ≠ 0; *z* > 1.96, *p* < .05). The model structures and the individual factorial weights can be seen in Fig. 4, Fig. 5. We also replicated the CFAs considering only formally diagnosed participants (*n* = 165; Supplemental Table 1), for both long and short versions of the PANAS-FA. Once again, results indicated a good model fit for both versions: Long, CFI = .98; TLI = .98, RMSEA = .042 [IC90% .023-.057]; Short, CFI = .99; TLI = .98, RMSEA = .046 [IC90% .005-.071].

### Reliability

We assessed the reliability level of the long and short versions of the PANAS-FA, using McDonald's omega (ω) and Cronbach's alpha (α). Results were acceptable for both positive (Long, ω = .90, α = .89; Short, ω and α = .87) and negative (Long, ω and α = .92; Short, ω and α = .90) factors.^35^ Similar results were also seen for the whole measure (Long, ω and α = .89; Short, ω and α = .86).

### Convergent validity

Next, we examined the convergent validity of the PANAS-FA, checking how its factors correlate with general anxiety and the need for affect. As shown in Table 2, the negative factor of PANAS-FA was significantly associated with both general anxiety (GAD-7) and the emotional avoidance factor.

**Table 2:** PANAS-FA factors correlations with anxiety and Need for affect.

	Anxiety GAD-7	Emotion Approach	Emotion Avoidance
Positive (Long)	-.001	.115	.005
Positive (Short)	.014	.099	-.016
Negative (Long)	.370∗	.018	.404∗
Negative (Short)	.362∗	.013	.392∗

Note: ∗*p* < .001

### Mediation

Finally, based on the correlational results, we assessed whether PANAS-FA's negative factor (short) could predict general anxiety, mediated by emotion avoidance. In Step 1, we assessed the total effect, with the negative factor significantly predicting general anxiety (X → Y; B = .40, IC95% = .237-0.549, *p* < .001). In Step 2, we assessed the indirect effect, or the influence of negative emotions on general anxiety, through emotion avoidance (X → M → Y; B = .15, IC95% = .088-.229*, p* < .001). Finally (Step 3) we assessed the direct effect of negative emotions on general anxiety, after the inclusion of the mediator (B = .25, IC95% = .078-.414, *p* < .001). These results indicate a partial mediation, as the influence of X on Y remained significant after the inclusion of the mediator. Thus, negative affect influences general anxiety, partially influenced by emotion avoidance.

### Group differences

Finally, we performed group differences in the positive and negative emotions regarding participants' gender (Men n = 63; Women n = 142), whether they were prescribed an auto-injector, and between single and multiple FAs. No significant differences (*p* > .05) were found in participants' gender, in neither the full or short versions of the PANAS-FA dimensions. However, these differences were significant when considering whether they were prescribed an auto-injector (Yes n = 58; No n = 147), with individuals that require its use presenting higher positive (Long version: Yes = 2.03, No = 1.75*; t*[203] = 2.51, *p* < .05; Short version: Yes = 2.39, No = 2.08; *t*[203] = 2.18, *p* < .05) and negative emotions (Long version: Yes = 2.10, No = 1.68; *t*[203] = 3.50, *p* < .001; Short version: Yes = 2.30, No = 1.83; *t*[203] = 3.35, *p* < .001). Finally, we assessed the differences on positive and negative emotions considering whether the participants have one (n = 121) or multiple (n = 84) food allergies. Multiple allergies' individuals presented significantly higher results in both positive (Long version: Multiple = 1.97, Single = 1.72; *t*[203] = −2.41, *p* < .05; Short version: Multiple = 2.38, Single = 2.01; *t*[203] = −2.82, *p* < .01) and negative emotions (Long version: Multiple = 1.99, Single = 1.67; *t*[203] = −2.86, *p* < .01; Short version: Multiple = 2.22, Single = 1.79; *t*[203] = −3.41, *p* < .001).

## General discussion

Because of the growing prevalence of food allergy and its adverse impact on health-related quality of life, closer attention is needed to the psychological distress specific to the condition. However, reliable and valid measures are required to ensure the confidence in measurement, results, and any consequent decision making. The present research aimed to adapt the Positive and Negative Affect Schedule into an FA-specific emotions tool, the PANAS-FA, assessing its structure and validity through a range of powerful techniques.

Health outcomes researchers are increasingly involved in questionnaire development, evaluation, and refinement. For multi-item scales, Item Response Theory (IRT) methods provide a clear picture of the performance of each item (question) in the scale and how the scale functions overall for measuring the construct of interest in the study population, leading to short reliable questionnaires that are tailored to the population of interest. Initially, we assessed the parameters of PANAS-FA items using IRT (discrimination, difficulty, and information). All items presented high or very high levels of discrimination,^37^ in addition to contributing a considerable information level to the total score of their respective factors. However, when assessing item difficulty, 4 items of each factor presented values out of the recommended threshold.^38^ When assessing the content of these 4 items, such results seemed reasonable in that it is unlikely that having FA would generate excitement or enthusiasm in an individual. However, as the items presented other good parameters, we decided to test 2 versions of the PANAS-FA, 1 considering all 20 items and 1 considering the 12 best items.

We tested the structure of these 2 models using Confirmatory Factor Analysis. Results showed a good (and similar) fit for both models. That is, their two-dimensional structure is robust and suitable to assess FA-specific emotions. Moreover, they also presented acceptable internal consistency levels^35^ for their isolated factors and the full measures. Such robust results attest that both versions of the PANAS-FA, with 20 and 12 items, can be used in research. However, as the 20-item version includes eight items that might not contribute to the overall emotional impact in FA, the shorter measure might be preferable and present more accurate results. We then replicated the CFAs considering only individuals formally diagnosed with an FA, and once again, the results showed a good model fit.

Moreover, we tested the convergent validity of the measures, checking how PANAS-FA factors are related to general anxiety and the need for affect. Anxiety can be defined as an emotional response that includes feelings like tension, uncertainty, nervousness, and worry,^42^ which are all negative responses. Our results showed a significant association between general anxiety and the negative factor of the PANAS-FA. This factor was also significantly associated with the emotion avoidance factor of need for affect, representing how much an individual avoids emotion-inducing situations.^27^ Such findings suggest that individuals with a higher incidence of negative emotions would also attempt to avoid engaging in situations that might bring with them the possible experience of new emotions. To further assess these relations, we decided to test whether the negative pole of PANAS-FA would explain general anxiety, as mediated by emotion avoidance. Results indicated significant partial mediation. In other words, negative emotions influence the level of general anxiety, partially influenced by emotion avoidance. Such associations can provide important insights for medical and psychological clinicians. Individuals experiencing strong negative emotions as a result of their FA might feel the need to seclude themselves to avoid experiencing new situations, which in turn can result in higher anxiety. Therefore, clinicians would likely need targeted techniques to help patients reduce the occurrence of negative emotions and assist them in engaging in new activities (eg, going to outdoor meals), which can help reduce the negative perception of the disease.

Finally, we tested group differences regarding participants' gender, the requirement of an auto-injector (or not), and single vs multiple food allergies. No significant differences were found regarding participants' gender, suggesting that men and women present similar levels of positive and negative emotions when focusing on their food allergies. Moreover, as stated in Supplemental Table 1, our sample comprises 58 (28.3%) individuals who were prescribed an auto-injector. Because of this specific group's higher risks, it is relevant to understand whether they experience emotions differently from those who do not need to use the device. Therefore, we assessed the differences regarding their emotions. The auto-injector group presented significantly higher levels of both positive and negative emotions, indicating that this specific group experience more strongly the emotions originated from their FA. Such findings might suggest that they may feel more alert or attentive (positive emotions) regarding their food allergy due to its risks and feel more scared or upset (negative emotions) about living with it. These groups differences are also significant when comparing individuals with one FA with those who have multiple. As for the auto-injector group, those with multiple FA might be at higher risk, as they have higher restrictions when compared to those that need to be on alert to just a single food type. As a result, they experience emotions more strongly when focusing on their FAs.

### Limitations, future studies, and implications

We would also like to note some limitations that attach to the present study. First, the use of a convenience sample restricting the possibility of generalization of our findings. Second, the sample originated exclusively from the United Kingdom, not assessing whether the structure holds cross-culturally. Third, we did not control whether participants presented an IgE-mediated FA or not, considering everyone that reported at least one type of FA (165 formally diagnosed). However, if the respondents perceive that they have a FA, they must also avoid the allergen and behave as if they have allergy, regardless of their true allergic status. The psychosocial impact will therefore also be similar. Also, we highlight that the goal of this paper was to adapt the PANAS-FA and assess its item parameters, model structure, reliability, and validity, and this goal was successfully achieved. It is unlikely that these limitations would influence our findings. Finally, we used a general anxiety measure, which does not control for the influence of other social (eg, daily stressors) or healthy factors (eg, co-morbidities). Instead, an FA-focused anxiety measure is preferable, such as the Food Allergy Anxiety Scale.^43^

Future studies should assess whether the structure of PANAS-FA is invariant across countries. Such studies could also assess whether the 12 items considered the “best” in our study are replicated in different cultures or populations, or whether any differences influence which emotions are affected by food allergy. Moreover, studies could use the PANAS-FA to understand better the associations between specific FA emotions and FAQL, along with other relevant and related factors such as subjective happiness, optimism or type of coping or management. Identifying which FA emotions are related to these factors may be vital for future interventions, providing an environment that focuses on or promotes particular emotions that can enhance individual well-being. Also, future studies can further assess the emotional differences between individuals who have been prescribed an auto-injector and those who do not have to use it or individuals with single and multiple FAs. In our findings, those with an auto-injector or multiple FAs presented significantly higher positive and negative emotions. Understanding the socio-psychological mechanisms that lead individuals to feel these emotions more strongly can help develop more positive experiences while reducing the negative sensations. Future studies could also assess the differences between those with IgE-mediated and non-IgE-mediated FA. Finally, researchers could also expand the findings of our mediational model. We have found that negative affect influences general anxiety and that this influence happens partially because of emotional avoidance. Thus, focusing on helping individuals constructively deal with emotions may support more positive outcomes across various factors.

In conclusion, our findings may also contribute value to the clinical setting. Consider, for instance, Cognitive Behavioural Therapy (CBT), which proposes a cognitive change.^44^ Identifying the most prominent emotions that arise from the food allergy can lead to specific strategies to improve the FA quality of life. That is, therapists can assist address and adjust the cognition, which can help reduce the negative emotions and improve the positive ones. CBT has been widely used to help regulate emotions in individuals facing different problems, such as alcohol dependency,^45^ major depressive disorders,^46^ and social anxiety disorder.^47^ Finally, we are confident that adopting the Positive and Negative Affect Schedule focused on food allergy (PANAS-FA) provides a novel opportunity to understand the intrinsic associations between emotions and living and coping with FA.

## Abbreviations

Food Allergy Quality of Life, (FAQL); Positive and Negative Affect Schedule, (PANAS).

## Ethical approval

All procedures performed in this study involving human participants were in accordance with the 1975 Helsinki Declaration. This study was approved by the ethics committee of the School of Applied Psychology, University College Cork.

## Informed consent

Informed consent was obtained from all participants.

## Consent for publication

All authors provided input into the manuscript, reviewed the final draft, and provided consent for publication.

## Authors contributions

Drs Gabriel Coelho and Audrey DunnGalvin wrote the first draft of the manuscript, which was then reviewed, amended, and approved by all co-authors.

## Funding

This project is funded through the 10.13039/100014364National Children's Research Centre, Paediatric Research Project Grants 2018 (C/18/12).

## Data availability

Derived data supporting the findings of this study are available from the corresponding author upon request.

## Declaration of competing interest

The authors have no conflict of interest to declare.
