# Supplementary material for: The Positive and Negative Affect Schedule — Food Allergy (PANAS-FA): Adaptation and psychometric properties
Source: World Allergy Organ J. 2021 Dec 5;14(12):100615. doi: 10.1016/j.waojou.2021.100615 (PMC8654619; doi:10.1016/j.waojou.2021.100615)
Supplement: Multimedia component 1 [file mmc1.docx]

| **Supplemental Table 1**  *Allergy Characteristics of Sample* | | |
| --- | --- | --- |
| *Type of Allergy* | *n* | % |
| Cow's Milk | 40 | 19.5% |
| Eggs | 22 | 10.7% |
| Tree Nuts | 43 | 21% |
| Peanuts | 58 | 28.3% |
| Shellfish | 45 | 22% |
| Wheat | 13 | 6.3% |
| Soy | 11 | 5.4% |
| Fish | 20 | 9.8% |
| Other | 79 | 38.5% |
| *When diagnosed* |  |  |
| Between 2005 and 2019 | 89 | 43.8% |
| Before 2005 | 114 | 56.2% |
| *Diagnosed by* |  |  |
| Allergist | 42 | 20.5% |
| Paediatric Specialist | 11 | 5.4% |
| General Practitioner \ Family Doctor | 93 | 45.4% |
| Allergy Nurse | 6 | 2.9% |
| Dietician | 8 | 3.9% |
| Alternative Practitioner | 1 | 0.5% |
| Not formally diagnosed | 40 | 19.5% |
| Other | 4 | 2% |
| *Prescribed auto-injector* |  |  |
| Yes | 58 | 28.3% |
| No | 147 | 71.7% |
